# The Effect of Neoadjuvant Therapy on Early Complications of Esophageal Cancer Surgery

**Published:** 2015-07

**Authors:** Mohammadtaghi Rajabi Mashhadi, Reza Bagheri, Abbas Abdollahi, Mohammad Javad Ghamari, Soudabeh Shahidsales, Maryam Salehi, Reza Shahkaram, Mohamad Reza Majidi, Shima Sheibani

**Affiliations:** 1 *Endoscopic & Minimally Invasive Surgery Research Center, Ghaem Hospital, Faculty of Medicine, Mashhad University of Medical Sciences, Mashhad, Iran.*; 2 *Cardio-Thoracic Surgery & Transplant Research Center, Emam Reza Hospital, Faculty of Medicine, Mashhad University of Medical Sciences, Mashhad, Iran.*; 3 *Surgical Oncology Research Center, Mashhad University of Medical Sciences, Mashhad, Iran.*; 4 *Department of Radiotherapy & oncology, Ghaem Hospital, Faculty of Medicine, Mashhad University of Medical Sciences, Mashhad, Iran.*; 5 *Department of Community Medicine, Faculty of Medicine, Mashhad University of Medical Sciences, Mashhad, Iran.*; 6 *Sinus and Surgical Endoscopic Research Center, Ghaem Hospital, Faculty of Medicine, Mashhad University of Medical Sciences, Mashhad, Iran.*

**Keywords:** Esophageal Cancer, Neoadjuvant Therapy, Surgery

## Abstract

**Introduction:**

Early diagnosis and appropriate treatment is required in esophageal cancer due to its invasive nature. The aim of this study was to evaluate early post-esophagectomy complications in patients with esophageal cancer who received neoadjuvant chemoradiotherapy (NACR).

**Materials and Methods:**

This randomized clinical trial was carried out between 2009 and 2011. Patients with lower-third esophageal cancer were randomly assigned to one of two groups. The first group consisted of 50 patients receiving standard chemoradiotherapy (Group A) and then undergoing surgery, and the second group consisted of 50 patients undergoing surgery only (Group B). Patients were evaluated with respect to age, gender, clinical symptoms, type of pathology, time of surgery, perioperative blood loss, and number of lymph nodes resected as well as early post-operative complicate including leakage at the anastomosis site, chylothorax and pulmonary complications, hospitalization period, and mortality rate within the first 30 days after surgery.

**Results:**

The mean age of patients was 55 years. Seventy-two patients had squamous cell carcinoma (SCC) and 28 patients had adenocarcinoma (ACC). There was no significant difference between the two groups with respect to age, gender, time of surgery, complications including anastomotic leakage, chylothorax, pulmonary complications, cardiac complications, deep venous thrombosis (DVT), or mortality. However, there was a significant difference between the two groups regarding hospital stay, time of surgery, perioperative blood loss, and number of lymph nodes resected.

**Conclusion:**

The use of NACR did not increase early post-operative complications or mortality among patients with esophageal cancer.

## Introduction

Esophageal cancer is the ninth most common cancer in the world and the fifth most common in developing countries (1). Despite considerable treatment successes, esophageal cancer is still associated with a poor prognosis (2). Various methods and techniques have been proposed for the treatment of esophageal cancer, yet there is no consensus on the best treatment. The preferred treatment in localized and resectable esophageal cancer is esophagectomy, a procedure which can be performed in different ways (3). However, local or systemic relapses are common after surgery and the 5-year survival rate varies between 10-39% (4,5). The presence of micrometastases in local lymph nodes or distant organs is considered a main prognostic factor for post-operative mortality (6). 

The key focus in the treatment of this cancer is thus to diagnose such micrometastases prior to surgery and to prevent the formation of macrometas- tases (7).

Chemoradiotherapy along with surgery leads to good outcomes in the treatment of esophageal cancer, including decreasing the stage of the disease, increasing the chance of cancer eradication, and also elimination of micrometastases (8,9). 

A number of studies have been reported regarding the benefits of surgery followed by chemoradiotherapy as a neoadjuvant treatment for the management of esophageal cancer (10-12). Evidence suggests that this approach may result in increased patient survival rates (13-15). 

However, neoadjuvant treatment has been associated with early post-operative side effects and complications (16). Such early side effects may be divided into local and systemic complications, including leakage at the anastomosis site, formation of chylothorax, pulmonary complications, wound infection, mediastinal adherence, and also increased difficulty in tumor removal and increased mortality within 30 days after surgery (17).

The goal of this study was to evaluate early post-operative side effects of esophagec- tomy among two groups of patients: those undergoing surgery followed by neoadjuvant chemoradiotherapy (NACR) and those undergoing surgery with no NACR.

## Materials and Methods

This was a randomized clinical trial carried out between 2009 and 2011 in Ghaem and Omid hospitals, Mashhad, both affiliated to Mashhad University of Medical Sciences. One-hundred patients suffering from esophageal cancer were included in the study. Inclusion criteria were (1) lower esophageal cancer; (2) general condition suitable for surgery, as well as lack of previous cardiac, pulmonary, or renal problems; (3) no contraindication to neoadjuvant treatment; and (4) lack of distant macroscopic metastases. 

Exclusion criteria included (1) cervical, upper, and middle-part esophageal cancer; (2) no desire for surgery following NACR; (3) intolerance to surgery after receiving NACR; (4) acute malnutrition (albumin< 2.5g/dl); (5) macrometastases (Stage 4); and (6) serious complication during surgery such as airway damage or intense bleeding.

Preoperative staging was performed in all patients, including a laboratory examination, endoscopic ultrasound scan (EUS) and a computed tomography (CT) scan of the thorax and upper abdomen, as well as abdominal sonography and barium swallow. Because of local limitations, it was not possible to perform a positron emission tomography (PET) scan. Patients were randomly assigned to one of two groups using computer-generated random numbers. Group A included 50 patients receiving chemoradiotherapy and cisplatin, followed by 50 Gy radiation and then undergoing surgery 3–4 weeks later. The proximal field of radiation therapy was 5–7 cm to the tumor and the distal field was adjacent to L_1_. Group B included 50 patients undergoing surgery only.

The radiation technique consisted of initial anterior and posterior opposed fields at 4,000 cGy. On the first and final days of radiotherapy, patients received chemothera- py with cisplatin (20 mg/m^2)^ and 5-fluorouracil (5FU) (700 mg/m^2^/infusion over 24 hours). 

This regimen was administered in all patients who underwent transhiatal esophagectomy, and the stomach was used as a conduit. Anastomosis performed in the neck by hand.

Epidemiological and clinical information including age, gender, clinical symptoms, type of pathology, time of surgery, perioperative blood loss, number of lymph nodes resected, and early post-operative side effects including leakage at the anastomosis site, pulmonary complications (pneumonia, atelectasis, empyema, and pulmonary insufficiency), chylothorax, resectability of the tumor, hospitalization period, and mortality rate within the first 30 days after surgery were recorded in all patients and then analyzed by SPSS16 on an intention-to-treat basis. 

Categorical variables were compared between the two groups using the chi-square test or Fisher exact test as appropriate, whereas continuous variables were compared using Student’s t-test. Differences were considered to be statistically significant for P<0.05.

## Results

The age of patients ranged from 48–63 years (mean, 55 years). The mean age was 56.0±5.62 years in the group receiving NACR plus surgery and 57.7±3.80 years in the surgery-only group. There was no significant difference between the two groups in terms of age, gender, preoperative staging, or type of pathology. 

There was no significant difference in gender between the two groups (P=0.841) (Table 1 shows the patient characteristics). The mean hospitalization period was 12.26±1.084 days for Group A and 11.02±1.450 days for Group B. There was a significant difference between the two groups in terms of hospitalization period (P=0.001). 

Anastomosis site leakage was detected in none of the patients in the group receiving NACR plus surgery and one patient in the surgery-only group, although the difference was not statistically significant (P>0.05). 

Pulmonary complications such as atelectasia, pneumonia, empyema, and pulmonary insufficiency were observed in four patients in each of the groups, with no significant difference between the two groups (P>0.99). Chylothorax was observed in two cases in Group A and one case in Group B (P>0.99). In Group A, five patients developed post-operative cardiovascular accidents (myocardial infarction [MI] in three patients and arrhythmia requiring treatment in two patients). 

In Group B, six patients showed complications (three cases of MI and three cases of arrhythmia). 

Two patients from Group A and three patients from Group B developed deep vein thrombosis (DVT) and underwent appropriate treatment. In the first 30 days after surgery, mortalities occurred in four patients in the chemoradiotherapy plus surgery group (two patients due to MI complications and two patients due to pulmonary embolism) and in three patients in the surgery group (two patients due to extensive MI and one patient due to pulmonary embolism). 

There was no significant difference between the two groups (P=1.00). Significant differences between the two groups were identified in terms of time of surgery, perioperative blood loss, and number of lymph nodes resected (Table.1).

**Table 1:** Characteristics of study groups

**Parameter**		**Group A**	**Group B**		**P-value**
Number (Total=100)	Stage I II III IV	50	50		
Male: female		27/23	26/24		0.841
Type or pathology (ACC/SCC)		15/35	13/37		0.758
Preoperative clinical staging		Number 0 36 14 0		Number 1 34 15 0	0.05
Anastomotic leakage		0	1		0.05
Pulmonary complications		4	4		0.99
Cardiovascular complications		5	6		0.99
DVT and related complications		2	3		0.99
Chylothorax		2	1		0.99
Duration of hospitalization (days)		12.26±1.084	11.02±1.450		0.001
Hospital mortalities		4	3		0.99
Blood loss in the surgery		405 cc ± 25	390 cc ± 15		0.001
Time of surgery		185 _min_ ± 20	175 min ± 25		0.05
Number of lymph nodes		5 ± 2	7 ± 2		0.001

DVT: deep vein thrombosis

## Discussion

Esophageal cancer is an invasive condition associated with high rates of lymph node involvement and vascular invasion (6). As an invasive malignant condition, the disease requires very careful management. NACR along with esophagectomy may increase patients’ survival rate (4). Furthermore, use of NACR may improve local control of esophageal cancer after surgery (18). In this study, 100 patients with lower esophageal SCC were included, 50 of whom received NACR and then underwent transhiatal esophagectomy after 3–4 weeks and 50 of whom underwent transhiatal esophagectomy without NACR. Factors such as age, gender, tumor location, and clinical stage were evenly balanced between the groups. The mean hospitalization period was 12.26±1.084 days in the group with NACR and 11.02±1.450 days in the other group; a difference that was statistically significant. 

In a study reported by Berger et al (2005) in 179 patients suffering from esophageal cancer, the hospitalization period was the same in both groups (14). Other studies by Slater et al (2001) and Van Hagen et al (2012) reported similar results (19,20). In a study by Jones (1977) in 166 patients with esophageal cancer, the overall toxicity and side effects of NACR were very small and not clinically relevant (15).

In this study there was no significant difference between the two groups with respect to post-operative side effects, such as anastomosis site leakage, pulmonary complications, or wound infection. In a study by Bagheri et al (2011), a group of 40 patients with esophageal cancer receiving NACR were examined and no pulmonary complications were reported (8). In the study by Van Hagen et al (2012), a group of 366 patients suffering from esophageal cancer were examined and it was noted that only hematological side effects (neutropenia and leucopenia) and general side effects associated with chemoradiotherapy such as lack of appetite and fatigue were higher in the group receiving NACR than the other group, while early post-operative side effects such as anastomosis site leakage, cardiopulmonary complications and wound infection were the same in both groups (19). Also, in studies by Doty et al (2002) and Slater et al (2011) it was shown that early post-operative side effects in patients receiving NACR did not increase in comparison with the control group (20, 21). In this study, chylothorax was not significantly different in the group receiving NACR compared with the comparator group. In another study by Roul et al (2007) in 818 patients suffering from esophageal cancer, it was reported that early side effects such as anastomosis site leakage, wound infection, and chylothorax did not increase in the patients receiving NACR. On the other hand, cardiac complications such as arrhythmia and MI increased significantly in this group (17). 

Furthermore, in a study by Berger (2005), there was no significant difference between the two groups in terms of cervical anastomosis site leakage, pneumonia, acute respiratory distress syndrome (ARDS), chylothorax, arrhythmias and wound infection after surgery; only emergence of DVT was higher in the group receiving NACR (14). In this study, mortalities during the first 30 days after surgery included eight patients from the NACR group and six from the group undergoing surgery only, with no significant difference. Thus NACR does not cause early mortality after esophagectomy. In most studies, such as those reported by Heise (2001) , Berger (2005), and Van Hagen et al (2012), the use of NACR did not cause an increase in mortality rates after surgery (14,19,22).

There was no significant difference between the groups receiving or not receiving NACR in terms of early side effects of transhiatal esophagectomy for esophageal SCC. Only the emergence of chylothorax in the group receiving NACR was higher. Therefore, the use of NACR does not cause an increase in early post-operative complications.

## Conclusion

The use of NACR did not increase early post-operative complications or mortality among patients with esophageal cancer.
